# COVID-19 Concerns Among Old Age Psychiatric In- and Out-Patients and the Employees Caring for Them, a Preliminary Study

**DOI:** 10.3389/fpsyt.2020.576935

**Published:** 2020-10-30

**Authors:** Maria Stylianou Korsnes, Elsa Grødal, Elisabeth Kjellén, Tone M. C. Kaspersen, Kristin B. Gjellesvik, Jūratė Šaltytė Benth, Bodil A. McPherson

**Affiliations:** ^1^Department of Old Age Psychiatry, Oslo University Hospital, Oslo, Norway; ^2^Department of Psychology, University of Oslo, Oslo, Norway; ^3^Institute of Clinical Medicine, Ahus, University of Oslo, Oslo, Norway; ^4^Health Services Research Unit, Akershus University Hospital, Nordbyhagen, Norway

**Keywords:** COVID-19, inpatients, outpatients, employees, fear, psychiatry, hospitalization

## Abstract

A questionnaire was administered to 14 patients admitted at the Department of Old Age Psychiatric 24-h unit at Oslo University Hospital with questions about experiences and fears regarding COVID-19. A similar adjusted questionnaire was administered to 19 outpatients. The purpose was to investigate if the patients had fears, anxieties, and quality of life issues related to COVID-19 that could affect their treatment. A quest back questionnaire with similar questions about patient care and work conditions was sent to the personnel working with these patients, and 46 of 81 responded. Most patients welcomed the strict measures that were applied, including a visitation ban for inpatients and a reduction in consultations for the outpatients. Most patients reported that they were not very scared of getting COVID-19, nor did many believe that they would die if they were infected. A minority of patients reported being very worried. The patients also differed on other issues related to the COVID-19 situation. A minority were negative to the interventions, rules, and regulations, and/or considered the risk of infection to be elevated at the clinic, and/or that the quality of their daily life was negatively impacted. Employees more often than patients were concerned about the COVID-19 influence on their health. They were also concerned about being at work amid the crisis. About half of their comments were related to the fear of inadvertently infecting patients with COVID-19. Also, a majority complained about aspects related to the implemented COVID-19 guidelines. This study is explorative in nature, mainly due to its small sample size, which makes it difficult to draw conclusions from the results. However, the results imply a need for addressing the COVID-19 concerns of both patients and employees, to prevent potential negative effects on treatment and overall life quality. Future research should investigate the self-reported effects of the pandemic situation on a larger sample size of elderly psychiatric patients.

## Introduction

The novel coronavirus disease (COVID-19) was first detected in Wuhan, China in December 2019. Within 2 months it was declared a Public Health Emergency of international concern by the World Health Organization (1) and by mid-June 2020 the disease has caused over 400,000 deaths globally (2). In Norway, the first case was registered at the end of February, and in the following month, extensive measures described as the toughest and most invasive since World War II were initiated by the Norwegian Government to prevent the virus from spreading, aiming to reduce the scope of social contact between people from different households (3).

Since the pandemic outbreak, concerns have been raised about the psychological consequences of the pandemic situation and the measures undertaken to some vulnerable groups of people (4), including the elderly population, and particularly older persons with health problems, including psychiatric disorders. High age has been established a core risk factor for severe disease (5), and many of the common somatic diseases among the elderly place them in a risk group of severe disease if they were to be infected. This risk was increasingly reported in media, with examples from care homes in other countries with terrible outcomes. Thus, most elderly were fully aware of the risk and did their best to abide by the strict new rules and regulations on shielding and social distancing (6). However, particularly inpatients could fully control their environment. Thus, fear for epidemic and pandemic outbreaks were possible triggers of elevated psychological stress and anxiety in the general population of elderly (7) and possibly to a higher extent for groups with anxiety, depression, and mental health illness in general. Concerns have been raised about the psychiatric disease as a factor for elevated risk of infection, elevated barriers in assessing health services, and additional worsening of psychiatric symptoms (8, 9). Thus, older persons with mental health issues are possible victims of the cumulative/additive risk when additionally, being defined as a high-risk group of developing severe disease or death.

Furthermore, elderly persons who live alone or at an inpatient clinic risk being victims of the negative consequences of measures aiming for social distancing. This also applies to inpatients who are not allowed to receive visitors, one of the measures to prevent the disease to enter the clinic. A recent review of the psychological impact of quarantine has concluded that quarantine can lead to altered levels of stress and symptoms of depression (10). As a response to the pandemic, the Norwegian government advised the elderly to self-isolate and closed down day centers and voluntary projects aiming to help the elderly. These are possible causes of increased loneliness in this group (4, 11), leading to an elevated risk of anxiety and depression (12, 13). Collectively, these measures are concerns that may affect elderly persons with psychiatric disorders in multiple ways, obliging health professionals to be aware of possible consequences for symptoms and needs for treatment. Also, fear of being exposed to COVID-19 may affect the treatment of patients negatively if they become preoccupied with the fear of disease.

This study aimed to investigate how in- and outpatients in an old age psychiatry unit, and the personnel caring for them, are affected by the COVID-19 outbreak, regarding fear of being infected, perceived consequences of the pandemic situation and measures undertaken on symptom severity and treatment. The goal was to use this information to develop and implement appropriate interventions within each group regarding fear, conformity to interventions, rules and regulations, risk evaluation, and the quality of daily life.

## Methods

### Questionnaire

We compiled a questionnaire with 13 statements regarding fear of being infected with COVID-19 (Q1, Q2, Q5), consequences of interventions, rules and regulations (Q3, Q7, Q10, Q11), risk evaluation (Q4, Q9, Q12), and consequences for daily life due to COVID-19 (Q6, Q8, Q13). The statements are listed in Table 1. The participants responded to each statement on a scale from 0 (agree) to 10 (disagree). The questionnaire was administered to inpatients and outpatients, and the personnel (employees) filled out a quest-back (14) form sent by e-mail to all employees with patient interaction. Some of the statements were slightly different for each group to be relevant to their situation. Six statements were identical for all groups. The data were collected from March to June. Similar restrictions were valid for the entire duration of testing.

**Table 1:** Statements in the questionnaires (Q1–Q13).

**Fear of infection with COVID-19**	
Q1	I'm afraid of being infected with COVID-19^a, b, c^
Q2	I'm scared to die if I get infected with COVID-19^a, b, c^
Q5	I feel that fear of getting COVID-19 makes me sicker^a, b^/is heavy on me^c^
**Consequences of interventions, rules, and regulations**	
Q3	I feel that the measures at the clinic^a, b^/workplace^c^ to prevent COVID-19 infection are too strict
Q7	I think the introduction of the visit ban^a^/measures to reduce infection due to COVID-19^b, c^ was/were correct
Q10	I was given sufficient information about the COVID-19 situation at hospitalization^a^/at the department^b^/at my workplace^c^
Q11	I think the clinic^a, b^/workplace^c^ guidelines to avoid infection were difficult to relate to
**Risk evaluation**	
Q4	I think the risk of infection is greater by being at the clinic^a, b^/at work^c^ than being at home
Q9	I have concerns about being hospitalized^a^/meeting at the clinic^b^/being at work^c^ due to the COVID-19 situation
Q12	I have taken other precautions myself to reduce the chances of getting infected^a, b, c^ (yes/no^a, b^)
**Consequences of COVID-19**	
Q6	I think my treatment at the clinic^a, b^/my working conditions^c^ has gotten worse because of COVID-19
Q8	I think the COVID-19 situation has affected my health^a, b, c^
Q13	I think the COVID-19 situation has adversely affected my improvement process^a, b^/health situation^c^ (yes/no^a, b^)

a *Inpatients.*

b *Outpatients = 17.*

c *Employees*.

A quest back option was ruled out for the patients, since they are of an age where the majority is not comfortable with using a computer, and since it was important to verify that they understood the questions correctly.

### Participants

The clinic provides inpatient and outpatient treatment for persons over the age of 65 with psychiatric symptoms. The current patient sample is typical. The patients received appropriate medication and treatment according to their condition, such as physiotherapy, psychotherapy, occupational therapy, environmental therapy, conversational therapy, and group therapy.

#### Inclusion Criteria

The employees responsible for the patient's diagnosis and treatment made sure the recruited patients were fit to answer the questions. Patients with severe symptoms of depression, anxiety, or cognitive impairment were excluded, as were patients with ongoing psychosis or mania that could have influenced their ability to understand and answer the questions. The inclusion criteria for employees invited to participate, was that they interacted with the patients on a daily basis.

#### Inpatients

The inpatients are elderly, over the age of 65 with psychiatric disorders who require 24-h care. They were referred for assessment and treatment, and hospitalization periods vary from days to weeks. Most patients are referred from their primary care doctor, and all participants were voluntary admitted. When not at the hospital, most live at home. Some live alone and some with partners. All of them filled out the questionnaire while being hospitalized. The additional measures imposed on them included strict sanitation rules, restricted or canceled group activities and walks, and importantly, a ban on all visitations.

#### Outpatients

The outpatients are elderly, over the age of 65 with psychiatric disorders. They filled out the questionnaire as part of their visit to the outpatient clinic. They were referred from their primary care doctor or receive follow up treatment. Note that only home-dwelling patients participated, since the patients living in nursing homes were quarantined and not able to participate. The additional measures imposed on them included strict sanitation rules, canceled consultations, and partly telephone consultations/video (Confrere) consultations. Those who asked were mostly allowed to come to the outpatient clinic for their consultations.

#### Employees

The employees working with inpatients were in the process of moving from the countryside into the city to be collocated with the outpatient clinic. This led to increased stress and uncertainty for the employees, which in turn causes an increased burden on them in addition to the concerns caused by the COVID-19 outbreak. All personnel working with the patients were invited to participate in the anonymous quest back poll, and about two-thirds responded. The personnel include nurses, physiotherapists, occupational therapists, psychiatrists, psychologists, and other health personnel.

### Comments

All patients could comment on their answers, and the comments are referred to when appropriate. The employees were only able to give a general comment at the end of the quest-back form.

### Ethics

The study was evaluated by the data protection office at Oslo University Hospital, and the conditions for the study were revised and explained. The study was labeled as a quality enhancement study.

The inpatients and outpatients were asked to fill out the form as honestly as possible, and were told by the experimenter that their responses would be anonymous and not to be shared with other personnel. Their responses were typed into an Excel sheet by an experimenter. A key code was created, and the key code was recorded on the sheet and in the Excel sheet. A separate paper that contains a link between the patients and the key codes are kept locked. The coded response sheets are kept locked in a separate location.

The employees filled out the quest-back form on their computer, and their responses are completely anonymous. There is no stored information linking each respondent to his/her responses.

### Analysis

Demographic characteristics and diagnostics of the inpatients and outpatients were presented as frequencies. Due to small group sizes, percentages were not presented. There was no demographic information about the employees registered, due to the complete anonymity of the survey. The main aim was to explore the within-group patterns, which were described by means and standard deviations (SDs) and medians and first and third quartiles. In addition, Spearman's correlation coefficients were calculated among statements covering the same topics. The overlapping statements (Q1, Q2, Q4, Q8, Q11, and Q12) were compared between the groups by Independent-Samples Median test or χ^2^-test, as appropriate. The descriptive statistics are shown in Table 2. In the case of significant overall differences, the pairwise comparisons were carried out with Bonferroni correction applied for each statement. The comments of the inpatients and outpatients were described. The statistical analyses were performed in SPSS v 26.

**Table 2:** Descriptive statistics for questions within groups.

	**Outpatients**	**Inpatients**	**Employees**	**χ^**2**^ (df)^**6**^**	***p*-value^**7**^**
	**(*N* = 19)**	**(*N* = 14)**	**(*N* = 46)**		
**Fear of infection with COVID-19**					
**Q1**					
Mean (SD)	4.7 (3.9)	5.9 (4.6)	5.9 (2.8)		
Median (Q_1_, Q_3_)	5 (0, 8)	8.5 (0, 10)	6 (3.8, 8)	0.93 (2)	0.628
**Q2**					
Mean (SD)	4.5 (3.2)	6.6 (4.5)	7.1 (3.2)		
Median (Q_1_, Q_3_)	5 (2, 7)	9.5 (0, 10)	8 (5, 10)	9.72 (2)^8^	**0.008**
**Q5**					
Mean (SD)	6.7 (3.6)	6.9 (3.4)	5.9 (2.9)		
Median (Q_1_, Q_3_)	8 (5, 10)	8 (4.8, 10)	5.5 (4, 9)		
**Consequences of interventions, rules and regulations**					
**Q3**					
Mean (SD)	7.1 (3.6)	8.7 (3.1)	8.4 (2.6)		
Median (Q_1_, Q_3_)	9 (5, 10)	10 (9.8, 10)	10 (7, 10)		
**Q7**					
Mean (SD)	1.9 (3.0)^*a*^	1.6 (3.1)	1.9 (2.3)		
Median (Q_1_, Q_3_)	1 (0, 2.5)^*a*^	0 (0, 2.3)	1 (1, 1)		
**Q10**					
Mean (SD)	4.2 (4.2)^*a*^	2.1 (3.2)	2.5 (2.8)		
Median (Q_1_, Q_3_)	3 (0, 8.3)^*a*^	0.5 (0, 3.3)	1 (1, 2)		
**Q11**					
Mean (SD)	8.2 (3.0)^*a*^	8.6 (2.7)	8.5 (2.3)		
Median (Q_1_, Q_3_)	10 (8, 10)^*a*^	10 (7.5, 10)	9 (8, 10)		
**Risk evaluation**					
**Q4**					
Mean (SD)	6.6 (3.9)	6.4 (4.1)	4.8 (3.4)		
Median (Q_1_, Q_3_)	9 (3, 10)	8 (3, 10)	4 (1, 8)	3.30 (2)	0.192
**Q9**					
Mean (SD)	9.1 (2.0)^*a*^	7.7 (3.7)^*c*^	7.4 (3.3)		
Median (Q_1_, Q_3_)	10 (8.8, 10)^*a*^	10 (5, 10)^*c*^	9 (5, 10)		
**Q12**					
Yes, *n*	15^*b*^	6	23^*d*^	7.34 (2)^*i*^	**0.025**
**Consequences of COVID-19**					
**Q6**					
Mean (SD)	7.4 (3.3)	9 (2.2)	5.0 (3.0)		
Median (Q_1_, Q_3_)	9 (5, 10)	10 (8.8, 10)	4 (3, 8)		
**Q8**					
Mean (SD)	5.8 (3.3)^*a*^	7.0 (3.7)	7.3 (2.9)		
Median (Q_1_, Q_3_)	5 (3, 9.3)^*a*^	9 (3.5, 10)	8 (5, 10)	2.70 (2)	0.259
**Q13**					
Yes, *n*	6^*a*^	1^*c*^	6^*e*^		

a *N = 18.*

b *N = 17.*

c *N = 13.*

d *N = 39.*

e *N = 36.*

f *Statistics for Independent-Samples Median test. (χ^b^) with degrees of freedom (df).*

g *p-Value for Independent-Samples Median test.*

h *χ^b^(1) = 8.53 (p = 0.010) for pairwise comparison of outpatients vs. employees, χ^b^(1) = 5.13 (p = 0.071) for pairwise comparison of outpatients vs. inpatients, and χ^b^(1) = 0.37 (p = 1.00) for pairwise comparison of inpatients vs. employees.*

i *χ^b^(1) = 4.65 (p = 0.093) for pairwise comparison of outpatients vs. employees, χ^b^(1) = 7.24 (p = 0.021) for pairwise comparison of outpatients vs. inpatients, and χ^b^(1) = 1.08 (p = 0.999) for pairwise comparison of inpatients vs. employees.*

*The bold value indicates significant (p < 0.05)*.

## Results

**Figure 1:**
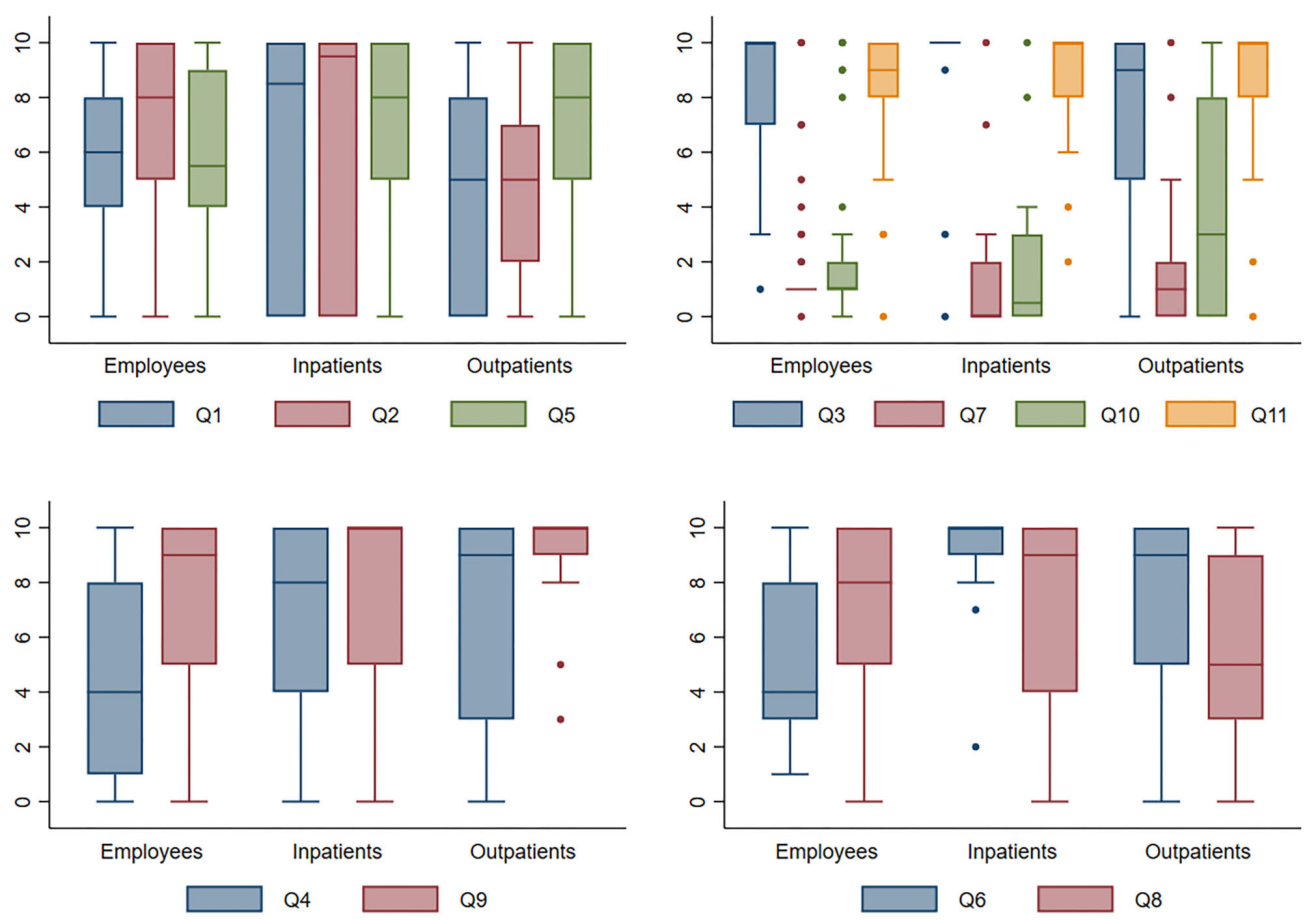
The boxes present median and first and third quartiles. The lines at the bottom/top vertical bars present adjacent values defined as the most extreme values within 1.5 × IQR, where IQR is interquartile range, of the nearer quartile. The dots present outliers. Q1–Q11 corresponds to the statements in the questionnaires.

The results are presented in Figure 1, see Table 3 for demographics and diagnostics.

**Table 3:** Demographic characteristics, and diagnostics.

	**Outpatients (19)**	**Inpatients (14)**
**Sex**		
Women	13	8
Men	6	6
**Age group**		
50–59 years	0	2
60–69 years	6	5
70–79 years	7	3
80–90 years	6	4
**Education level**		
<10 years	2	5
10–15 years	10	6
16–20 years	4	3
Ed. level not entered	3	
**Diagnosis (first and secondary)**		
Depression	15	12
Anxiety	1	6
Bipolar	0	2
Psychosis	0	2
Cognitive deficit/dementia	2	1
Diag. not entered	3	1

### Fear of Infection With COVID-19

Statements Q1, Q2, and Q5 explore whether the participants were afraid to become ill with COVID-19, if they were scared of death if they caught the disease, and if they thought that any fears about catching the disease would have a negative effect on their treatment, or for the employees, if it would cause a burden on them.

Most of the inpatients answered that they were not afraid of being infected (median 8.5), and if they were to be infected, most inpatients answered that they were not afraid to die from it (median 9.5) and that the fear of getting COVID-19 did not make them sicker (median 8). The correlation between statements Q2 and Q5 was very strong, while other correlations were weak. The outpatients were moderately afraid of being infected or die from COVID-19 (median 5, correlation 0.6). They also indicated that the fear of getting COVID-19 did not make them sicker (median 8). The employees were moderately afraid of being infected (median 6), but not afraid of dying if they catch the disease (median 8). They also meant that the fear does not impose much load on them (median 5.5). The answers of the employees correlated positively but only moderately. There were overall differences between the groups regarding statement Q2 (*p* = 0.008), with the outpatients significantly more afraid to die than employees (*p* = 0.010), with no differences between other groups.

Notably, the minority of inpatients were very concerned, but the majority of the comments to statement Q1 shows that many are unconcerned “do not think I will be infected,” “don't know anyone with the disease,” “don't want to think about it” (7/11 comments), while a minority is quite afraid “due to my age,” “I am old,” “I think all must be afraid” (4/11 comments). The outpatients gave similar comments: not scared “I don't care since I have cancer,” “I take precautions,” or scared “since I have an underlying condition,” “it's a terrible death.” Comments to statement Q2 show a mixture where some “are not afraid of death,” “think I will make it through,” while others are very afraid due to underlying conditions. Comments to statement Q5 are overwhelmingly from those who are unconcerned (6/7 comments from the inpatients and 1/2 of the comments from the outpatients).

### Consequences of Interventions, Rules, and Regulations

Statement Q3, Q7, Q10, and Q11 explore different aspects on how the interventions, rules, and regulations have been received and how easy they have been to relate to, and the perceived consequences of the measures taken.

Responses to statement Q7 show agreement among all participants that it was correct to implement strict measures to curb the spreading of COVID-19 to patients and personnel. The employees and the inpatients agreed that they have gotten enough information about the situation (statement Q10, median 1 and 0.5, respectively), while outpatients were slightly less satisfied with the information given to them (median 3). A large majority of the inpatients, outpatients and employees thought the measures had been appropriately strict (statement Q3), and it had not been difficult to relate to them (statement Q11). Among outpatients, the statements Q3 and Q7, and statements Q7 and Q11 correlated negatively moderately, while statements Q3 and Q11 correlated positively moderately. Moderate positive correlation was found between statements Q7 and Q10 among inpatients and negative moderate correlation between statements Q3 and Q7 among employees.

The inpatients' comments to statement Q3 showed overwhelming understanding for the measures taken at the hospital for inpatients (12/12 comments), and also from most outpatients (5/8 comments). A minority (3/8) outpatient complained about “teleconsultations,” “no group sessions,” and “postponement of meetings.” Most inpatients (6/7) commented to statement Q7 that the visitation ban was warranted, while one commented that “visitors should be able to come if they are not sick.” The outpatients had more general statements, but most comments were positive “feel taken care of,” “protect the elderly,” “thankful that precautions have been implemented.” Comments from the inpatients to statement Q10 show positivity to the information they got, while the two comments from the outpatients both complained about lack of information about COVID-19. All comments to statement Q11 are positive “straightforward,” “clear rules,” “boring but OK.”

### Risk Evaluation

Statements Q4, Q9, and Q12 assess how the patients and employees evaluate the risks involved in being at the hospital or outpatient clinic, and whether they have taken any additional precautions to stay safe.

Statement Q4 showed that most inpatients and outpatients think they are safe (median 8 and 9, respectively). Note that the outpatients did not meet at the outpatient clinic as frequently as they normally would do, but partly participated in the online session via telephone or videoconference. Employees think they are not as safe as the patients (median 4). Neither the patients nor the employees think the risk is higher at clinic/work than at home (statement Q9). Among the outpatients and inpatients, statements Q4 and Q9 correlated positively, but only moderately. Most outpatients have taken other precautions to avoid being infected (statement Q12), with the exception of approximately half the inpatients and employees. There were also overall differences between the groups regarding statement Q12 (*p* = 0.025), but the pairwise comparison showed only significant difference between inpatients and outpatients (*p* = 0.021).

Comments to statement Q4 show a mixed picture where all inpatient and outpatient comments on hygiene are positive, and the minority commented concern about meeting more people. Comments to statement Q9 show the same pattern of comments, most feel “safe” and “trust,” but a minority are concerned about “Increased risk of infection.” Comments to statement Q12 list the precautions they have done, including “washing hands,” “isolation,” “avoid visiting stores,” “gloves,” “mask,” “avoid public transport,” “follow government advises.”

### Consequences for Daily Life Due to COVID-19

Statements Q6, Q8, and Q19 probed how the participants believed that their daily life has been affected by the COVID-19 outbreak.

The majority of inpatients and outpatients did not think their treatment (statement Q6) were affected by COVID-19 (median 10 and 9, respectively), while the employees complained about their working conditions (median 4). The improvement process (statement Q13) seems to be moderately or little affected by the COVID-19 restrictions among both outpatients and inpatients. While the inpatients did not claim that the COVID-19 affected their health (statement Q8, median 9), the outpatients' did think that their health was more affected due to the COVID-19 situation (median 5). The employees did not think the COVID-19 had affected their health in a large degree (statement Q8, median 8), and only a minority think the restrictions affected their health situation (statement Q13). While the statements did not correlate among the employees, statement Q8 and Q13 among in- and outpatients, and statement Q6 and Q13 among inpatients were negatively moderately correlated. In addition, statements Q6 and Q8 were positively moderately correlated among inpatients.

Even though a majority of inpatients and outpatients thought that the COVID-19 situation had not adversely affected their healing process, only those who responded oppositely commented. The comments are all related to aggravation of health problems or fears. Comments to statement Q8 from inpatients are overwhelming that the situation has not affected their health, one commented that “there are less activity and more worries.” Comments from the outpatients are all from those who think the situation has affected their health, they complain about “negativity,” “isolation,” “lack of physiotherapy,” and “insecurity.”

### General Comments to the Questionnaire From the Employees

Only 15 participants gave general comments to the statements. Five commented that they were concerned that they involuntarily may infect the patients since they may have the disease without showing symptoms, four uttered criticism to how the crisis has been handled, two complained about general stress, two mentioned additional stressors (moving process), and one uttered fear of getting the disease.

## Discussion

The patients were in general satisfied with the COVID-19 specific measures, even though some of the measures were quite invasive. They perceive that the measures were in their best interests. Even though most patients coped fine with the initial COVID-19 situation, a minority were afraid of the prospect and consequences of getting the disease, or were negative to the interventions, rules, and regulations, or considered that the risk of infection was elevated at the clinic, or that their quality daily life had been reduced. These are particularly important issues that need to be addressed in the interaction with the patients.

The COVID-19 pandemic can place elders in a situation where social isolation is difficult to avoid, especially those whose main source of social contact is outside of their homes (4). The elderly patients living at home must make active choices and, for example, restrain from meeting grandchildren, receive less help and care, not traveling collectively, etc. At the same time, several facilities were closed down (senior center, fitness center, restaurants, events). Some adjust fine, but as the responses to the statements show, patients, as well as the population at large, are individuals. Psychiatric patients can be particularly vulnerable to the negative effects of the COVID-19 pandemic and lockdown, such as isolation. Research have shown that they experience a larger increase in psychiatric symptoms like anxiety, depression, stress and insomnia during the pandemic compared to healthy controls (15), as well as symptoms of COVID-19-related stress (16, 17). These concerns also apply to elderly patients with cognitive decline, as the shut-down of societal functions can deprive them of needed social support and practical resources from their surroundings or community (18). Still, according to the results in this preliminary study, most of the included patients did well. They were not particularly afraid of the virus, and they understood and accepted the measures introduced by their section. However, one cannot ignore that a minority of patients reported a lot of fear and worry, and those who thought the pandemic situation had a negative impact on their daily lives and their improvement process. Due to the limited sample size and the current methodology, we could not predict who these patients are. Also, we did not measure how important each topic was for the patients, but from the comments, we often see stronger opinions from those who disagree with the majority. Thus, it becomes necessary to include thoughts and experiences about COVID-19 in the individual treatment of all patients, and conduct individual interviews to identify the patients who are negatively affected. From there, measures can be introduced to help these patients individually in the best possible way.

An unforeseen result is that most employees seem to be more frightened and worried than most patients. Although many employees have not responded, it nevertheless shows that the employees feel uncertain about their responsibility to the patient and how the regulations should be interpreted. Several employees felt that their working day was negatively affected by the pandemic situation. Other research also suggest that patients are not the only ones affected by the COVID-19 pandemic. A study done in China found that medical health workers risk mental health problems like anxiety, obsessive-compulsive symptoms, depression, insomnia, and somatization (19). The researchers suspected that the medical health workers experienced psychosocial stress due to a high workload and an unsafe work environment where many lack knowledge about the virus and how to prevent infection. Uncertainty and risk were indeed part of the employees' experiences with the pandemic situation, reported in our study. They had to familiarize themselves with the many new guidelines and regulations and experienced uncertainty about the responsibility for avoiding the spread of infection and caring for a group of patients at risk. This suggests the importance of good dialogue about this in the workplace.

The study has several weaknesses. The sample size is limited and the results cannot be generalized outside one psychiatric clinic in Norway. The questionnaires had not been verified as research tools. Even though similar restrictions were in place for the entire duration of testing, some respondents responded early in the COVID-19 pandemic (March–April) when there was intense media focus and many were surely overwhelmed by the fierce measures and severity of the situation, while others responded in May after there has been a more positive focus in the Norwegian media.

Regardless of its limitations, the results of this study imply the COVID-19 pandemic impact individuals quite differently, both among elderly psychiatric patients and the employees working with them. Further research should therefore strive to gain more knowledge in this area, preferably by using a larger sample size. It is useful to clarify exactly what characterizes the elderly patients with the highest risk of adverse effects from the COVID-19 pandemic, and whether clinical or demographic information can help us identify the patients (and employees) in need of extra care and attention during the pandemic situation.

## Data Availability Statement

The raw data supporting the conclusions of this article will be made available by the authors, without undue reservation.

## Ethics Statement

Ethical approval was not provided for this study on human participants because this study is considered a quality enhancement study outside the mandate of the regional ethics committee. The patients/participants provided their written informed consent to participate in this study.

## Author Contributions

MK initiated the study, wrote the manuscript, and compiled the figures. EG developed the questionnaires and organized the quest-back form for the employees. EK contributed in writing the introduction and performs the literature search, gathered data from the outpatient clinic, and gathered consent forms from the outpatients, in addition she gave critical input on the interpretation of results and the discussion. TK administered the questionnaire and consent form for all inpatients, and made sure they understood the statement, she gathered and interpreted the comments from the inpatients. KG analyzed the data in SPSS, compiled Table 3, and contributed to the discussion. JB performed the group analysis in SPSS, compiled Table 1, and wrote the analysis and the results section of the manuscript. BM gave critical input from the initial planning until the final draft and contributed to the discussion and revised the document. All authors contributed to the article and approved the submitted version.

## Conflict of Interest

The authors declare that the research was conducted in the absence of any commercial or financial relationships that could be construed as a potential conflict of interest.
