# Supplementary material for: COVID-19 Concerns Among Old Age Psychiatric In- and Out-Patients and the Employees Caring for Them, a Preliminary Study
Source: Front Psychiatry. 2020 Oct 30;11:576935. doi: 10.3389/fpsyt.2020.576935 (PMC7673424; doi:10.3389/fpsyt.2020.576935)
Supplement: Supplementary file 1 [file Data_Sheet_1.PDF]

quest back - Employees (no personalia collected)

I am afraid of being contaminated by Covid-19	**scale 1-10**<br>1 - agree<br>10 - disagree	comment:
I am afraid of dying if I get contaminated by Covid-19	**scale 1-10**<br>1 - agree<br>10 - disagree	comment:
I believe the measures at my workplace to prevent Covid-19 contamination are too strict	**scale 1-10**<br>1 - agree<br>10 - disagree	comment:
I think that the risk of getting contaminated is greater being at work than staying at home	**scale 1-10**<br>1 - agree<br>10 - disagree	comment:
My fear of getting Covid-19 is a burden to me	**scale 1-10**<br>1 - agree<br>10 - disagree	comment:
I believe that my working situation at the department has become tougher because of Covid-19	**scale 1-10**<br>1 - agree<br>10 - disagree	comment:
I think the implemenation of measures to prevent Covid-19 contamination was right	**scale 1-10**<br>1 - agree<br>10 - disagree	comment:
I believe the Covid-19 situation has had an impact on my health	**scale 1-10**<br>1 - agree<br>10 - disagree	comment:
I have concerns about working at the Department of Old Age Psychiatry because of the Covid-19 situation	**scale 1-10**<br>1 - agree<br>10 - disagree	comment:
I receive sufficient information about the Covid-19 situation at Department of Old Age Psychiatry	**scale 1-10**<br>1 - agree<br>10 - disagree	comment:
I think the Department guidelines to prevent contamination are difficult to relate to	**scale 1-10**<br>1 - agree<br>10 - disagree	comment:
I have taken my own further precautions to reduce chances of contamination	**A:** yes<br>**B:** no	if yes, which:

I believe the Covid-19 situation has had a negative impact on my health	**A:** yes<br>**B:** no	If yes:<br>**A**: Worsening of previous ailments/ symptoms **B**: Getting new/ other ailments/ symptoms<br>**C:** Other

Andre kommentarer:
