# Supplementary material for: COVID-19 Concerns Among Old Age Psychiatric In- and Out-Patients and the Employees Caring for Them, a Preliminary Study
Source: Front Psychiatry. 2020 Oct 30;11:576935. doi: 10.3389/fpsyt.2020.576935 (PMC7673424; doi:10.3389/fpsyt.2020.576935)
Supplement: Supplementary file 3 [file Data_Sheet_3.PDF]

Oslo University Hospital

Oslo University hospital HF
Department of Mental Health
Clinic Mental health and addiction

Outpatient form

Survey: Covid-19 impact on patients and their interaction with Oslo University Hospital at Department of Old Age Psychiatry

Anonymous survey.

Identification code (not name)	Education (number of years)
Age	Diagnosis group (psychosis/dementia/depression/other)
Gender	Diagnosis / symptoms (free text)

1. I am afraid of getting contaminated by Covid-19

agree 0 1 2 3 4 5 6 7 8 9 10 disagree

If 0-1 / 9-10:
Comment:____

2. I am afraid of dying if I get contaminated by Covid-19

agree 0 1 2 3 4 5 6 7 8 9 10 disagree

If 0-1 / 9-10:
Comment:____

3. I believe that the outpatient clinic is less available because if the Covid-19 pandemic

agree 0 1 2 3 4 5 6 7 8 9 10 disagree

If 0-1 / 9-10:
Comment:____

4. I think that the risk of getting contaminated is greater meeting at the outpatient clinic than staying at home

agree 0 1 2 3 4 5 6 7 8 9 10 disagree

If 0-1 / 9-10:
Comment:____

5. I believe that my fear of getting Covid-19 makes me sicker

agree 0 1 2 3 4 5 6 7 8 9 10 disagree

If 0-1 / 9-10:
Comment:____

6. I think my treatment at the outpatient clinic has been poorer because of Covid-19

agree 0 1 2 3 4 5 6 7 8 9 10 disagree

If 0-1 / 9-10:
Comment:____

7. I believe the measures undertaken at the outpatient clinic to prevent Covid-19 have been correct

agree 0 1 2 3 4 5 6 7 8 9 10 disagree

If 0-1 / 9-10:

Comment:____

8. I believe the Covid-19 situation has had an impact on my health

agree 0 1 2 3 4 5 6 7 8 9 10 disagree

If 0-1 / 9-10:

Comment:____

9. I have concerns about meeting at the outpatient clinic because of the Covid-19 situation

agree 0 1 2 3 4 5 6 7 8 9 10 disagree

If 0-1 / 9-10:

Comment:____

10. I have recieved information about the Covid-19 situation at the clinic

agree 0 1 2 3 4 5 6 7 8 9 10 disagree

If 0-1 / 9-10:

Comment:____

11. I think the Department of Old Age Psychiatry guidelines to prevent contamination are difficult to relate to

agree 0 1 2 3 4 5 6 7 8 9 10 disagree

If 0-1 / 9-10:

Comment:____

12. I have taken my own further precautions to reduce chances of contamination

Yes / No

If yes: Which?____

13. I think the Covid-19 situation has had a negative impact on my recovery

Yes / No

If yes:

- Worsening of ailments Yes / No
- Prolonged period of illness Yes / No
- New symptoms Yes / No
- Other Yes / No

14. Other comments:

____

Thank you for contributing ☺
