# Supplementary material for: COVID-19 Concerns Among Old Age Psychiatric In- and Out-Patients and the Employees Caring for Them, a Preliminary Study
Source: Front Psychiatry. 2020 Oct 30;11:576935. doi: 10.3389/fpsyt.2020.576935 (PMC7673424; doi:10.3389/fpsyt.2020.576935)
Supplement: Supplementary file 4 [file Table_1.docx]

Supplementary table. Spearman’s correlations among variables within three groups

|  | Q2 | Q5 | Q7 | Q10 | Q11 | Q9 | Q12 | Q8 | Q13 |
| --- | --- | --- | --- | --- | --- | --- | --- | --- | --- |
| Outpatients | |  |  |  |  |  |  |  |  |
| Q1 | 0.55 | 0.02 |  |  |  |  |  |  |  |
| Q2 |  | -0.01 |  |  |  |  |  |  |  |
| Q5 |  |  |  |  |  |  |  |  |  |
| Q3 |  |  | -0.63 | 0.03 | 0.50 |  |  |  |  |
| Q7 |  |  |  | 0.21 | -0.66 |  |  |  |  |
| Q10 |  |  |  |  | -0.02 |  |  |  |  |
| Q11 |  |  |  |  |  |  |  |  |  |
| Q4 |  |  |  |  |  | 0.50 | -0.10 |  |  |
| Q9 |  |  |  |  |  |  | -0.20 |  |  |
| Q12 |  |  |  |  |  |  |  |  |  |
| Q6 |  |  |  |  |  |  |  | 0.05 | 0.26 |
| Q8 |  |  |  |  |  |  |  |  | -0.61 |
| Q13 |  |  |  |  |  |  |  |  |  |
| Inpatients | |  |  |  |  |  |  |  |  |
| Q1 | 0.99 | 0.33 |  |  |  |  |  |  |  |
| Q2 |  | 0.36 |  |  |  |  |  |  |  |
| Q5 |  |  |  |  |  |  |  |  |  |
| Q3 |  |  | -0.13 | 0.16 | 0.13 |  |  |  |  |
| Q7 |  |  |  | 0.71 | -0.40 |  |  |  |  |
| Q10 |  |  |  |  | -0.17 |  |  |  |  |
| Q11 |  |  |  |  |  |  |  |  |  |
| Q4 |  |  |  |  |  | 0.59 | -0.19 |  |  |
| Q9 |  |  |  |  |  |  | -0.08 |  |  |
| Q12 |  |  |  |  |  |  |  |  |  |
| Q6 |  |  |  |  |  |  |  | 0.65 | -0.57 |
| Q8 |  |  |  |  |  |  |  |  | 0.09 |
| Q13 |  |  |  |  |  |  |  |  |  |
| Employees | |  |  |  |  |  |  |  |  |
| Q1 | 0.45 | 0.57 |  |  |  |  |  |  |  |
| Q2 |  | 0.47 |  |  |  |  |  |  |  |
| Q5 |  |  |  |  |  |  |  |  |  |
| Q3 |  |  | -0.47 | 0.02 | 0.24 |  |  |  |  |
| Q7 |  |  |  | 0.13 | -0.24 |  |  |  |  |
| Q10 |  |  |  |  | -0.04 |  |  |  |  |
| Q11 |  |  |  |  |  |  |  |  |  |
| Q4 |  |  |  |  |  | 0.27 | -0.06 |  |  |
| Q9 |  |  |  |  |  |  | -0.30 |  |  |
| Q12 |  |  |  |  |  |  |  |  |  |
| Q6 |  |  |  |  |  |  |  | 0.07 | -0.27 |
| Q8 |  |  |  |  |  |  |  |  | -0.40 |
| Q13 |  |  |  |  |  |  |  |  |  |
